# The Anti-Aging Hormone Klotho Promotes Retinal Pigment Epithelium Cell Viability and Metabolism by Activating the AMPK/PGC-1α Pathway

**DOI:** 10.3390/antiox12020385

**Published:** 2023-02-05

**Authors:** Shuyan Zhou, Jacob Hum, Kaan Taskintuna, Stephanie Olaya, Jeremy Steinman, Junfeng Ma, Nady Golestaneh

**Affiliations:** 1 Department of Ophthalmology, Georgetown University Medical Center, Washington, DC 20057, USA; 2 Department of Oncology, Lombardi Comprehensive Cancer Center, Georgetown University Medical Center, Washington, DC 20057, USA; 3 Department of Neurology, Georgetown University Medical Center, Washington, DC 20057, USA; 4 Department of Biochemistry and Cellular & Molecular Biology, Georgetown University Medical Center, Washington, DC 20057, USA

**Keywords:** retinal pigment epithelium, retina, aging, PGC-1a, AMPK, Klotho, AMD

## Abstract

Initially discovered by Makuto Kuro-o in 1997, Klotho is a putative aging-suppressor gene when overexpressed and accelerates aging when deleted in mice. Previously, we showed that α-Klotho regulates retinal pigment epithelium (RPE) functions and protects against oxidative stress. However, the mechanisms by which Klotho influences RPE and retinal homeostasis remain elusive. Here, by performing a series of in vitro and in vivo experiments, we demonstrate that Klotho regulates cell viability under oxidative stress, mitochondrial gene expression and activity by inducing the phosphorylation of AMPK and p38MAPK, which in turn phosphorylate and activate CREB and ATF2, respectively, triggering PGC-1α transcription. The inhibition of Klotho in human RPE cells using CRISPR-Cas9 gene editing confirmed that a lack of Klotho negatively affects RPE functions, including mitochondrial activity and cell viability. Proteomic analyses showed that myelin sheath and mitochondrial-related proteins are downregulated in the RPE/retina of *Kl*^-/-^ compared to WT mice, further supporting our biochemical observations. We conclude that Klotho acts upstream of the AMPK/PGC-1α pathway and regulates RPE/retinal resistance to oxidative stress, mitochondrial function, and gene and protein expressions. Thus, KL decline during aging could negatively impact retinal health, inducing age-related retinal degeneration.

## 1. Introduction

Klotho (KL) was first identified in 1997 as an anti-aging gene since its mutation induces premature aging phenotypes in mice [1], reducing their lifespan to about 8–9 weeks.

The α-klotho protein is encoded by the KL gene and regulates phosphate/calcium homeostasis [2], transient receptor potential cation channel subfamily V member 5 (TRPV5) [3], oxidative stress responses [4] and vitamin D metabolism [5]. Additionally, the KL protein is involved in numerous intracellular signaling pathways, including fibroblast growth factor-23 [6], Wnt [7], cAMP [8,9], p53/p21 [10], PKC [11], transforming growth factor-β [12], insulin-like growth factor [13,14], mammalian target of rapamycin [15] and Na+ /K+ ATPase [16]. The *KL* gene is highly conserved in humans, mice, and rats, with 98% sequence homology between humans and mice [17]. The molecular mechanisms by which KL exerts its anti-aging and protective effects in different tissues have been investigated. In the kidney, KL alleviates diabetic nephropathy by activating LKB1-AMPK-PGC1α and mediating renal mitochondrial protection [18], and retards renal fibrosis by acting on mitochondrial dysfunction and cellular senescence in renal tubular cells [19]. The energy sensor AMPK is an enzyme that is activated by increases in the cellular AMP/ATP ratio and regulates autophagy through the direct phosphorylation of ULK1 [20,21]. The peroxisome proliferator-activated receptor-gamma coactivator (PGC)-1alpha (PGC-1α) is known as a master regulator of mitochondrial biogenesis [22]. It is also involved in adaptive thermogenesis, respiration, and oxidative metabolisms by regulating mitochondrial and nuclear genes [23,24,25]. PGC-1α also plays a role in autophagy/mitophagy [26,27] and the detoxification of oxidative stress [28] and is highly expressed in the retina [29]. Studies have shown that AMPK can increase PGC-1α expression and activation [30]. α-Klotho levels have been shown to decrease with aging in human serum [31], and its decline might contribute to age-related diseases, including lung disease [32], diabetes [33], heart failure [34] and kidney disease [35]. It could also be linked to an increased mortality rate in American adults [36]. Age-related decline in Klotho is also reported to induce mitochondrial dysfunction and impair muscle regeneration [37]. The retinal pigment epithelium (RPE) is a vital component of the retina and is essential for maintaining healthy visual function. The RPE consists of a pigmented monolayer of polarized cells and plays a crucial role in photoreceptor health and functions [38].

With aging, various morphological and functional changes have been reported in the RPE [39]. These include the decline in the RPE cells in the central portion of the macula [40], multinucleation of RPE cells along with an increase in their size [41], thickening of the RPE–Bruch’s membrane layer [42], accumulation of lipofuscin granules, disorganization of inner basal infolding [43,44], loss of melanin granules [45], impaired lysosomal [46] and mitochondrial functions [47,48,49]. While a vast body of evidence supports the changes to RPE morphology and function with aging, the underlying mechanisms behind these phenotypes remain elusive. Previously, we have shown that Klotho regulates RPE functions and protects against oxidative stress [50]. Another group also showed that Klotho is crucial to sustaining retinal function [51]. More recently, we showed that *Pgc-1α* repression and a high-fat diet could induce AMD-like phenotypes in mice and that the AMPK/PGC-1α pathway was dysfunctional in the RPE of AMD donor eyes [52,53]. However, how these pathways are regulated in the RPE/retina remains to be elucidated. Here, through a series of in vitro and in vivo experiments, we investigate the mechanisms underlying the protective effects of Klotho in the RPE and retina.

## 2. Materials and Methods

### 2.1. Cells

The human RPE (ARPE-19) cells were validated in the Molecular Mechanisms Section of the Laboratory of Retinal Cell and Molecular Biology (NIH/NEI) by the American Type Culture Collection (ATCC) Cell Line Authentication Service (Promega, Madison, WI, USA) using tandem repeat analysis. The Amelogenin gender-determining locus was a perfect match for the ATCC human cell line CRL-2302 (ARPE-19) [54]. ARPE19 cells were cultured daily in DMEM/F12 medium (Gibco, Waltham, MA, USA, 11320033) supplemented with 10% FBS (Gibco, 10438-026) and 2 mM GlutaMAX (Gibco, Waltham, MA, USA, 35650061). To analyze protein phosphorylation by KL, the cells were starved with DMEM/F12 medium, containing no serum and 2 mM GlutaMAX for 24 h followed by HBSS (Gibco, Waltham, MA, USA, 14025-092) incubation for 4 h. Fresh HBSS containing 10 nM recombinant human Klotho protein (R&D Systems, Minneapolis, MN, USA, 5334-KL-025) was added. Cells were harvested at 0 min, 15 min, 30 min, and 1 h after Klotho treatment and processed for protein extraction and Western blotting. For qPCR analyses, the ARPE19 cells were cultured in DMEM/F12 medium supplemented with 1% FBS, 2 mM GlutaMAX, and 10 nM Klotho for 48 h. Cells were cultured with the same conditions without Klotho as control. The media were changed every 24 h. Cells were then collected for qRT-PCR. 

### 2.2. Animals

All studies involving animals were approved by the Institutional Animal Care and Use Committee of Georgetown University. Klotho knockout (*Kl*^-/-^) on a C57BL/6 background were obtained as a gift from Dr. Makoto Kuro-o (University of Texas Southwestern Medical Center, Dallas, TX, USA). *Kl*^-/-^ and wildtype (WT) mice on the same background were euthanized at 8 weeks of age. Both male and female mice were used in this study. RPE/retina were isolated for further experiments.

### 2.3. Cell Viability Assay

ARPE19 cells (1.4 × 10^4^ cells/well) were cultured in 96-well plates (Corning, Durham, NC, USA, 3603) for 48 h, with different concentrations of H_2_O_2_ (0–1 mM), in the presence or absence of 10 nM human Klotho protein for 24 h or 48 h. Cell viability was measured following 30 min of incubation with PrestoBlue Cell Viability Reagent (Invitrogen, Carlsbad, CA, USA, A13261). Fluorescence emission was measured at λex/em = 560 nm/590 nm.

### 2.4. qRT-PCR

The total RNA was extracted from ARPE19 cells or RPE/retina of mice by the E.Z.N.A. total RNA kit I (Omega BIO-TEK, Norcross, GA, USA, 6834-01) and reverse-transcribed with Verso cDNA synthesis kit (Thermo Fisher Scientific, Waltham, MA, USA, AB1453B). A total of 10 μg cDNA from each sample was used for each qPCR reaction using Maxima SYBR Green/ROX qPCR Master Mix (2X) (Thermo Fisher Scientific, Waltham, MA, USA, K0222). Specific primers (Table S1) for each gene were designed with the PrimerBank (https://pga.mgh.harvard.edu/primerbank/, accessed on 5 July 2022) and synthesized by Integrated DNA Technologies (IDT). 

### 2.5. Western Blotting

The cells or mice retina/RPE were lysed using T-PER tissue protein extraction reagent (Thermo Fisher Scientific, Waltham, MA, USA, 78510) plus protease and phosphatase inhibitor (Thermo Fisher Scientific, Waltham, MA, USA, A32959). Protein concentrations were measured by BCA assay kit (Thermo Fisher Scientific, Waltham, MA, USA, 23227). Protein samples were analyzed by the NuPAGE electrophoresis (Invitrogen) and transferred onto the PVDF membrane (Millipore, Rockville, MD, USA, IPVH00005) for immunoblotting. The target proteins were detected by the corresponding primary and secondary antibodies (Table S2). 

### 2.6. Complex I Activity Assay

Cells or mice retina/RPE were lysed and homogenized on ice with T-PER buffer containing protease and phosphatase inhibitor. The supernatant was collected by centrifugation at 16,000× *g* and 4 °C for 20 min. The protein concentration was determined by BCA assay kit, and 60 μg protein from mice tissue or 130 μg protein from cells was used to perform experiments. The complex I activity was measured using a Complex I Enzyme Activity Kit (Abcam, Waltham, MA, USA, ab109721). The absorbance was read at 450 nm in a transparent flat-well plate at 60 s intervals for 30 min. The activity of complex I was presented as rate/slope (mOD/min) per 60 μg protein lysate (from animal tissue) or 130 μg protein lysate (from cells).

### 2.7. mtDNA Copy Number

Mice RPE/retina or ARPE19 CRISPR/Cas9-*KL* KO cells were collected for DNA extraction by DNeasy Blood & Tissue Kit (Qiagen, Germantown, MD, USA, 69504). The mitochondrial DNA (mtDNA) gene *Nd1* (for mice tissue) and *ND1* (for human ARPE19 cells) and the nuclear DNA (nDNA) gene *H19* (for mice tissue) and *ACTB* (for human ARPE19 cells) were amplified by qPCR (CFX Connect Real-Time PCR Detection System). A 10 μL reaction was formulated by 5 μL PowerUp SYBR Green Master Mix (Thermo Fisher Scientific, Waltham, MA, USA, A25742), 3 μL template DNA, and 330 nM primers. The qPCR reaction was initiated at 50 °C for 2 min, followed by 95 °C for 2 min, then 40 cycles at 95 °C for 15 s and 60 °C for 1 min. All genes were run in triplicate. Cycle threshold values were analyzed to determine the relative ratio of mtDNA to nuclear DNA for each sample.

### 2.8. ROS Assay

The retina/RPE was dissected and homogenized on ice in 120 μL of RIPA buffer containing protease and phosphatase inhibitors. The concentration of lysate protein was measured using the BCA assay kit. The ROS levels were determined with the In Vitro ROS/RNS Assay Kit (Cell Biolabs, San Diego, CA, USA, STA-347) according to the manufacturer’s protocol. Fluorescence was read at 𝛌ex/em = 480 nm/530 nm using 96-well black-bottom flat plates (Greiner bio-one, Monroe, NC, USA, 655086). The ROS levels were presented as μM per 10 μg of protein.

### 2.9. CRISPR-Cas9 Gene Editing and Inhibition of KL

CRISPR/Cas9-mediated gene knockout (KO) technology was used in the ARPE19 cells to generate *KL* KO cells. Specific guide RNAs (sgRNAs) were designed using the following link https://zlab.bio/guide-design-resources (accessed on 30 November 2021) and ligated into LentiCRISPR v2 (ATCC, Plasmid #87360) according to the protocol listed on ATCC resource information section. The gRNAs used in this study were: Klotho sgRNA1 (5′–3′): GCGTTCCGGGAGTCTCCCGG; and Klotho sgRNA2 (5′–3′): CATCGACAACCCCTACGTGG. Cells transfected with control (scramble) or sgRNA were selected in medium with 2 μg/mL puromycin for 2–3 weeks, then 2 μM doxycycline (DOX) was added for 48 h to induce Cas9-eGFP expression. The ratio of the Cas9 positive cells was analyzed by flow cytometry.

### 2.10. NanoUPLC-MS/MS-Based Proteomics 

Mice retina were lysed using T-PER tissue protein extraction reagent (supplemented with protease and phosphatase inhibitors) as mentioned previously. Equal amounts of total proteins were used for quantitative proteomics by using similar procedures as those from our previous reports [55,56]. Briefly, proteins were digested by sequencing grade Trypsin/Lys-C (Promega) on suspension trapping columns, with the eluted peptides dried in a SpeedVac. Peptides were analyzed by a nanoAcquity UPLC system (Waters) interfaced with an Orbitrap Fusion Lumos mass spectrometer (Thermo Fisher Scientific, Waltham, MA, USA) in data-independent acquisition (DIA) mode. The label-free DIA data were processed with the Spectronaut v14 (Biognosys, Schlieren, Switzerland) in the hydridDIA mode with the mouse database downloaded from UniProt. Unpaired two-tailed Student’s *t*-test was used to define differentially expressed proteins (with fold changes >1.5 or <0.67; adjusted *p* values < 0.05). Volcano plot and gene ontology (GO) enrichment analysis were performed as described previously. 

### 2.11. Statistical Analysis

GraphPad Prism 8.3.0 software was used for all data analyses. Unpaired *t*-test was performed for comparison between two groups. Data are presented as mean ± SD or mean ± SEM from three independent experiments. Significance was considered when *p* ≤ 0.05 and was indicated in the text as follows: * p < 0.05, ** p < 0.01, and *** p < 0.001.

## 3. Results

### 3.1. KL Protein Protects RPE Cells from Oxidative Stress and Upregulates the Expression of Mitochondrial-Related Genes

**Figure 1:**
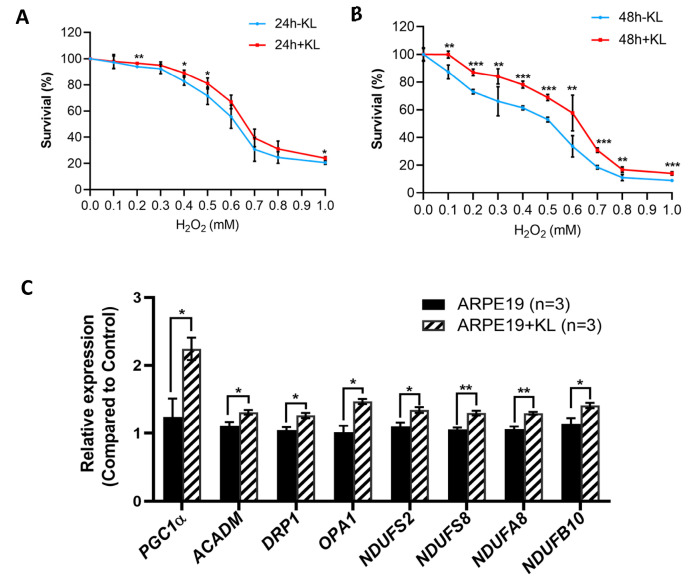
KL protein protects RPE from oxidative stress and upregulates the expression of genes related to mitochondrial activity. Cell viability assays of ARPE19 treated with increasing concentrations of H_2_O_2_ for 24 h (**A**) or 48 h (**B**) with or without 10 nM recombinant KL protein. Addition of KL for 48 h significantly increased the cell viability of ARPE19 at all H_2_O_2_ concentrations compared to control (without KL). The mean ± SD and the *p*-values were calculated for statistical significance. Holm–Sidak *t*-test was performed using GraphPad Prism 8.3.0. * p ≤ 0.05, ** p ≤ 0.01, *** p ≤ 0.001. (**C**) qPCR analyses of ARPE19 incubated with recombinant KL for 48 h revealed an increase in the expression of *PGC1α* (related to mitochondrial biogenesis, regulation of antioxidant genes and energy metabolism), *ACADM* (downstream target of PGC-1α, required by the initial step of fatty acid beta-oxidation), *DRP1* and *OPA1* (associated with mitochondrial fission and fusion), and respiratory chain complex I subunits (*NDUFS2, NDUFS8, NDUFA8, NDUFB10*), as compared to control. Graph represents mean ± SEM of three independent experiments. Holm–Sidak *t*-test was performed using GraphPad Prism 8.3.0. * *p* ≤ 0.05, ** p ≤ 0.01.

To test the effect of the anti-aging protein KL on the RPE cells’ viability, we exposed the ARPE19 to oxidative stress conditions established in our lab with increasing H_2_O_2_ concentrations for 24 and 48 h in the presence and absence of 10 nM recombinant KL protein. Our data showed that KL was able to protect ARPE19 from oxidative-stress-induced cell death both at 24 h and 48 h (Figure 1A,B). However, 48 h of oxidative stress combined with KL protein showed significant differences even at higher H_2_O_2_ concentrations between the KL-treated and -untreated cells (Figure 1B). Since mitochondria play an important role in oxidative stress, we analyzed the effect of KL protein on the expression of genes related to mitochondrial activity. The qPCR analyses of the cells treated with the recombinant KL for 48 h revealed an increased expression of genes related to mitochondrial activity, including *PGC1α* (mitochondrial biogenesis, regulation of antioxidant genes and energy metabolism), *ACADM* (required for the initial step of fatty acid beta-oxidation), *DRP1* (associated with mitochondrial fission), *OPA1* (plays a key role in mitochondrial fusion), and respiratory chain complex I subunits (*NDUFS2, NDUFS8, NDUFA8, NDUFB10*). Our data showed that KL was able to significantly increase the expression of the above genes in the ARPE19 cells, compared to the controls (Figure 1C).

### 3.2. KL Protein Regulates PGC-1α Expression in RPE by Inducing the Phosphorylation of AMPK, p38MAPK, ATF2/7, and CREB

**Figure 2:**
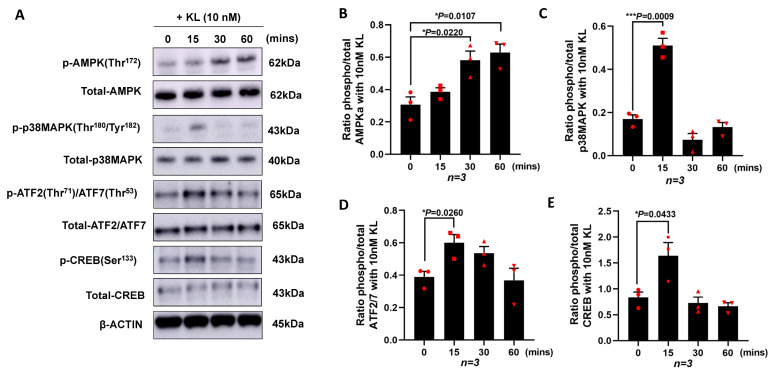
KL protein promotes the transcription of PGC-1α in RPE by increasing the phosphorylation of AMPK, p38MAPK, ATF2/7, and CREB. ARPE19 cells were cultured and starved for 24 h prior to being treated with 10 nM of recombinant KL protein for 0, 15, 30, and 60 min. (**A**) Western blots showing the phosphorylation of AMPK at Thr172, p38MAPK at Thr180/Tyr182, ATF2/ATF7 at Thr71/Thr53 and CREB at Ser133 at different time points. Total protein levels are shown for normalization. β-actin was used for internal control. (**B**–**E**) Relative expression of phosphorylated protein and total protein for each specific target compared with control (0 min, without KL), as determined by densitometry analysis of three independent Western blots. The relative densities of the bands were analyzed using ImageJ software. Unpaired *t*-test was performed using GraphPad Prism 8.3.0. Graph represents mean ± SEM, n = 3, * p ≤ 0.05, *** p ≤ 0.001.

To investigate the mechanisms by which KL regulates the expression of *PGC-1α* and the downstream genes, we cultured ARPE19 without serum in the presence of 10 nM of KL protein for different time points (0, 15, 30, and 60 min). The cells were lysed followed by protein extractions and Western blot analyses for various proteins. As shown in Figure 2A, the KL protein was able to induce the phosphorylation of AMPK at Thr172, p38 MAPK at Thr180/Tyr182, ATF2/ATF7 at Thr71/Thr53, and CREB at Ser133, compared to the untreated control cells (0 time point). The densitometry analysis of three independent Western blots showed a significant increase in pAMPK at 30 and 60 min of the KL treatment compared to the untreated cells (Figure 2B), as well as an increase in p-p38MAPK (Figure 2C), p-ATF2/7 (Figure 2D), and p-CREB (Figure 2E) at 15 min of the KL treatment as compared to the untreated control cells. AMPK can phosphorylate and activate CREB [57], which in turn activates the CRE sequence of the *PGC-1α* promoter [58]. In addition, p38MAPK phosphorylates ATF2, which in turn can activate the same CRE site, inducing the transcriptional activation of *PGC-1α*, as shown in Figure 1C.

### 3.3. KL Deficiency Induces Downregulation of Mitochondrial Proteins in the RPE/Retina of Mice

**Figure 3:**
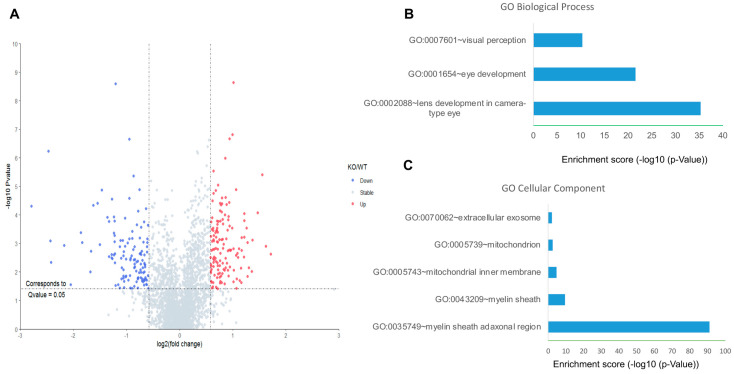
Proteomic analyses of the RPE/retina of WT and *Kl*^-/-^ mice. (**A**) The volcano plot for the retina proteome in WT and *Kl*^-/-^ mice. Blue color dots show down-regulated proteins, and red dots represent up-regulated proteins in *Kl*^-/-^ compared to the WT retina; GO cellular component (**B**) and biological process (**C**) of down-regulated retina proteins in the RPE/retina of WT and *Kl*^-/-^ mice (n = 5).

We used *Kl*^-/-^ and WT mice to further investigate the effects of KL on downstream pathways. First, unbiased quantitative proteomics was performed to explore the protein level changes in the retina of the WT and *Kl*^-/-^ mice. In total, 2700 retina proteins were identified and quantified. Among them, 263 proteins were significantly changed. More specifically, 138 proteins and 125 proteins were up-regulated and down-regulated, respectively (Figure 3A). Interestingly, the up-regulated proteins did not seem to be significantly enriched in specific clusters. In contrast, the down-regulated proteins were significantly enriched in biological processes, including lens/eye development and visual perception (Figure 3B). Moreover, highly enriched proteins appeared to be in the myelin sheath and mitochondria (Figure 3C). Down-regulated mitochondrial proteins indicated deregulated mitochondrial functions in the retina of the *Kl*^-/-^ mice.

### 3.4. KL Deficiency Induces Reduced AMPK, PGC-1α and DRP1 Protein, and Increased mTOR in the RPE/Retina of Kl^-/-^ Mice

**Figure 4:**
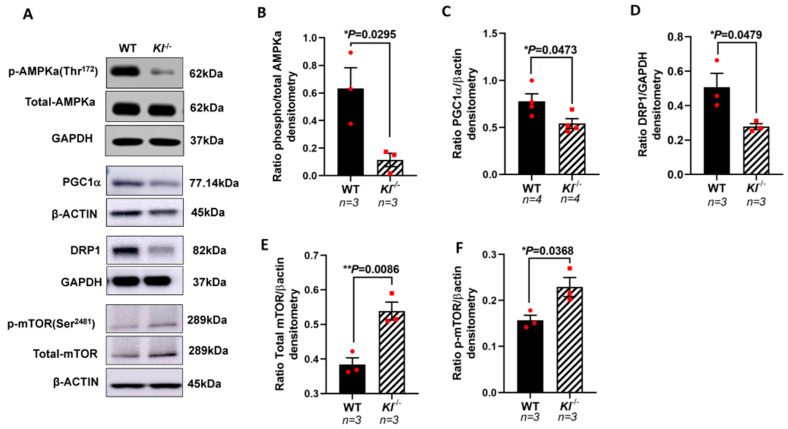
Reduced AMPK, PGC-1α, and DRP1 proteins, and increased mTOR protein expression in the RPE/retina of *Kl*^-/-^ mice. (**A**) Immunoblots of total AMPK and phosphorylated AMPK (Thr172), PGC-1α, DRP1, mTOR and phosphorylated mTOR (Ser2481) in control (WT) and *Kl*^-/-^ mice RPE/retina. (**B**) The ratios of phospho-AMPK/total AMPK were determined using densitometry analyses, showing decreased AMPK activity in the RPE/retina of *Kl*^-/-^ mice. (**C**,**D**) The PGC-1α and DRP1 levels normalized to β-actin and GAPDH, respectively, were significantly reduced in the *Kl*^-/-^ mice, as compared to WT mice. (**E**,**F**) Both total mTOR level and phosphorylated mTOR (Ser2481) levels were increased in the RPE/retina of *Kl*^-/-^ mice. The β-actin levels were used for normalization. Densitometry analyses were performed using ImageJ software. The unpaired *t*-test was performed using GraphPad Prism 8.3.0. Graph represents mean ± SEM, * *p* ≤ 0.05, ** *p* ≤ 0.01. n = 3~4, both male and female mice were included in each group.

Based on our in vitro observations, we further investigated the regulatory role of KL on the AMPK and PGC-1α pathway in vivo. We analyzed the phosphorylated levels of AMPK at Thr172 that are required for AMPK activation in the RPE/retina of the *Kl*^-/-^ and WT mice. Our data showed reduced p-AMPK (Thr172), suggesting an inactive AMPK in the RPE/retina of *Kl*^-/-^ compared to that of the WT mice (Figure 4A,B). We further tested the levels of PGC-1α protein and observed a decrease in its protein expression in the RPE/retina of *Kl*^-/-^ as compared to that of the WT mice (Figure 4A,C). An analysis of the DRP1 protein, the mitochondrial fission regulator, showed a decrease in the DRP1 protein levels in the RPE/retina of *Kl*^-/-^ as compared to those of the WT mice (Figure 4A,D). Because mTOR inhibits AMPK and studies have shown that increased mTOR activity can induce degeneration in the RPE [59], we measured the mTOR and p-mTOR protein levels in the RPE/retina of *Kl*^-/-^ compared to WT mice. Our data showed an increase in total mTOR and p-mTOR protein levels in the RPE/retina of *Kl*^-/-^ compared to WT mice, suggesting an overactive mTOR in *Kl*^-/-^ mice (Figure 4A,E,F). 

### 3.5. Decreased Mitochondrial Activity, mtDNA Copy Numbers, and Elevated ROS Levels in the RPE/Retina of Kl^-/-^ Mice

**Figure 5:**
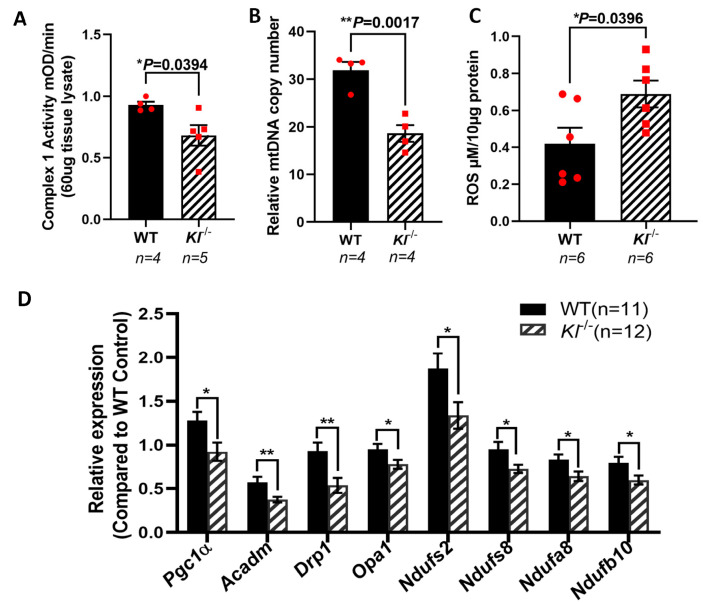
Reduced mitochondrial activity and elevated ROS in the RPE/retina of *Kl*^-/-^ mice. (**A**) Respiratory chain complex I activity is reduced in the RPE/retina of *Kl*^-/-^ mice, as compared to that of the control. (**B**) The mtDNA copy number was measured in the RPE/retina of *Kl*^-/-^ and WT mice (n = 4) by qPCR, showing significantly reduced mtDNA in the RPE/retina of *Kl*^-/-^ as compared to WT mice. (**C**) ROS production was significantly higher in the RPE/retina of *Kl*^-/-^ mice. (**D**) The expression of mitochondrial activity-associated genes *Pgc-1α*, *Acadm*, *Drp1*, *Opa1*, *Ndufs2*, *Ndufs8*, *Ndufa8*, and *Ndufb10* was decreased in the RPE/Retina of *Kl*^-/-^ mice as compared to WT mice. Graph represents mean ± SEM (WT group n = 11, 5 male and 6 female mice; *Kl*^-/-^ group n = 12, 6 male and 6 female mice). The unpaired *t*-test was performed using GraphPad Prism 8.3.0. * p ≤ 0.05, ** p ≤ 0.01.

KL is shown to regulate oxidative stress by activating FoxO forkhead transcription factors and the inhibition of IGF-1 [60]. PGC-1α plays a crucial role in sustaining the mitochondrial antioxidant defense [61] and upregulates the expression of ROS detoxifying enzymes [62,63]. To further explore the effects of KL on mitochondria, we measured the activity of the respiratory chain Complex I, and found that its activity is reduced in the RPE/retina of the *Kl*^-/-^ as compared to that of WT mice (Figure 5A). To determine the effect of KL deficiency on mitochondrial function, we measured the mtDNA copy numbers in the RPE/retina of the *Kl*^-/-^ and WT mice, and found significantly reduced mtDNA in the RPE/retina of the *Kl*^-/-^ mice as compared to that of the WT mice (Figure 5B). These observations support the regulatory role of KL in mitochondrial health and function. We also evaluated the ROS levels in the RPE/retina of the *Kl*^-/-^ mice as compared to that of the WT mice. Our data revealed increased levels of ROS in the RPE/retina of the *Kl*^-/-^ mice as compared to those of the WT mice (Figure 5C). qPCR analyses of mitochondrial-associated genes revealed a significant decrease in their expression in the RPE/retina of the *Kl*^-/-^ mice as compared to that of the WT mice (Figure 5D), further supporting our in vitro observations in Figure 1C and our total proteomic data in Figure 3C. 

### 3.6. Inhibition of KL by CRISPR-Cas9 Gene Editing in Human RPE Increased the Susceptibility to Oxidative Stress, Reduced Mitochondrial Activity and the Expression of Mitochondrial-Related Genes

**Figure 6:**
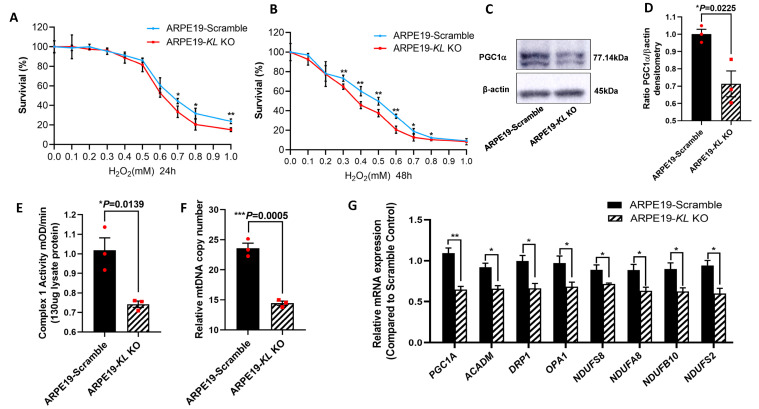
Inhibition of *KL* in human RPE reduces the cell viability and mitochondrial activity. ARPE19-CRISPR/Cas9-*KL* KO cells were treated with increasing concentrations of H_2_O_2_ for 24 h (**A**) or 48 h (**B**), followed by cell viability analyses. Cell viability was significantly higher in the control as compared to that of the ARPE19-*KL* KO cells at high concentration of H_2_O_2_ after 24 h of H_2_O_2_ treatment. Longer exposure to oxidative stress (48 h, H_2_O_2_) showed significant differences between the ARPE19-*KL* KO and control cells for 0.3–1 mM of H_2_O_2_. The mean ± SD and the *p*-values were calculated for statistical significance. Holm–Sidak *t*-test was performed using GraphPad Prism 8.3.0. * p ≤ 0.05, ** p ≤ 0.01. (**C**,**D**) PGC1 α protein levels were significantly lowered by inhibition of *KL* in ARPE19 cells for 4 weeks. (**D**) Densitometry analyses were performed by ImageJ software. (**E**) Mitochondrial complex1 activity was significantly reduced in the ARPE19-*KL* KO as compared to that in the scrambled control cells. (**F**) The mtDNA copy number was measured in the ARPE19-*KL* KO cells (n = 3) by qPCR, showing it was significantly reduced as compared to that of the control. (**G**) mRNA expression levels of mitochondrial activity-related genes, *PGC-1α*, *ACADM*, *DRP1*, *OPA1*, *NDUFS2*, *NDUFS8*, *NDUFA8,* and *NDUFB10* were significantly decreased after inhibiting the *KL* in ARPE19 cells for 3 weeks. The unpaired *t*-test was performed using GraphPad Prism 8.3.0. Graph represents mean ± SEM, n = 3, * *p* ≤ 0.05, ** *p* ≤ 0.01, *** *p* ≤ 0.001.

To further investigate the protective effect of KL on RPE cells, we generated Doxycycline (Dox) inducible *KL* knockout (KO) ARPE19 cells using CRISPR-Cas9 gene editing. The addition of 2 μM of Dox for 48 h induced GFP expression, indicating the expression of Cas9 protein (Supplementary Figure S1A). The ratio of Cas9-expressing cells was measured by flow cytometry, showing ~90% GFP-positive cells (Supplementary Figure S1B). The inhibition of KL protein expression was confirmed by Western blot and densitometry analyses and was 66% (Supplementary Figure S1C,D). ARPE19-CRISPR/Cas9- *KL* KO (ARPE19-*KL* KO) cells were analyzed for their susceptibility to oxidative-stress-induced cell death. The cells were incubated under increasing concentrations of H_2_O_2_ under our established stress conditions. Our data showed that ARPE19-*KL* KO cells have increased susceptibility to oxidative stress, and lower cell viability both at 24 h and 48 h of H_2_O_2_ incubation as compared to that of control cells with scrambled gRNA (Figure 6A,B). We also measured the protein expression of PGC-1α in the ARPE19-*KL* KO and control cells after 4 weeks of Dox induction. Western blot and densitometry analyses revealed a significant reduction in the PGC-1α protein levels in the ARPE19-*KL* KO compared to those of the control cells (Figure 6C,D). To confirm the effect of KL on mitochondrial health and function, we measured the Complex I activity in the ARPE19-*KL* KO and control cells. Our data showed reduced Complex I activity in the ARPE19-*KL* KO compared to that of the control cells (Fig6-E). qPCR analyses of the mtDNA further demonstrated mitochondrial dysfunction in the ARPE19-*KL* KO as compared to the control cells (Figure 6F). To complete our in vitro analyses, we measured the expression of mitochondrial-related genes by qPCR. Our data showed a significant decrease in the expression of mitochondrial-associated genes in the ARPE19-*KL* KO as compared to the control cells (Figure 6G).

## 4. Discussion

KL has been shown to regulate various cellular processes, including phosphate/calcium homeostasis [2], TRPV5 channels [3], oxidative stress responses [4], and vitamin D metabolism [5]. KL binds to the FGF23 receptor, a bone-derived hormone, with the ability to suppress phosphate reabsorption and Vit D production in the kidney [64]. Conversely, it has been shown that phosphate and vitamin D can regulate FGF23 and Klotho production, influencing their functions [65]. KL has also been shown to regulate various signaling pathways, such as Wnt [7], cAMP [8,9], p53/p21 [10], PKC [11], TGF-β [12], IGF-1 [13,14], mTOR [15] and Na+/K+ ATPase [16]. Thus, Kl plays an important role in cellular homeostasis and metabolic activities. 

Previously, we showed that KL was crucial for the regulation of RPE functions and protection against oxidative stress [50]. Simultaneously, another paper reported on Klotho as a crucial protein to sustain retinal function [51]. However, the mechanisms underlying KL actions in the RPE and retina remain elusive. 

Here, using a series of in vitro and in vivo analyses, we sought to investigate the mechanisms by which KL can influence RPE and retinal functions and maintain their homeostasis and metabolic activities.

Klotho has been reported to protect against oxidative stress [60]. Using ARPE19 cells under our established oxidative stress conditions with increasing H_2_O_2_ concentrations, we showed that the addition of KL protein could protect the cells from oxidative-stress-induced cell death both under 24 and 48 h of H_2_O_2_ and KL incubation. However, longer incubation times showed protections at all concentrations of H_2_O_2_, indicating that prolonged KL treatment can further increase cellular resistance to oxidative stress. To investigate the mechanisms by which KL could exert this protective effect, we analyzed the expression of the genes responsible for mitochondrial biogenesis, the regulation of antioxidant enzymes and energy metabolism, as well as genes of mitochondrial complex I activity. Our data showed that KL was able to induce the expression of the above genes, thus protecting the RPE cells against oxidative-induced cell death. Our data further support the previous observations on the role of KL and mitochondrial function [37]. 

To further analyze the signaling pathways by which KL influences cell viability and cellular metabolisms, we tested the effect of recombinant KL on ARPE19 cells in a time-course study. Western blot and densitometry analyses revealed that KL was capable of phosphorylating and activating AMPK, p-38MAPK, ATF2/7 and CREB. AMPK acts upstream of CREB and phosphorylates CREB on Ser133. p-38MAPK phosphorylates ATF2, an antiapoptotic protein that promotes cell survival. Activated CREB and ATF2 both act on the same CRE, inducing the transcription of *PGC-1α* and downstream genes. Studies have shown that both AMPK and p-38MAPK can induce *PGC-1α* during high-intensity exercise by activating CREB and ATF2 [66]. Interestingly, KL also seems to act upstream of *PGC-1α* to regulate RPE and retinal functions. The mechanism of function of Klotho as a hormone is poorly understood since no receptor has been identified to mediate its function. It is unknown how Klotho can induce protein phosphorylation. However, it is reported that the Klotho protein binds to FGF receptors (FGFRs). The complex Klotho–FGFR has a higher affinity to bind to FGF23 than FGFR or Klotho alone, enhancing the ability of FGF23 to induce the phosphorylation of FGF receptor substrate and ERK in different cell types [67,68].

Based on our in vitro data, we used *Kl*^-/-^ and WT mice to analyze the effect of KL on the RPE/retina in vivo. Total proteomics revealed the down-regulation of a number of highly enriched mitochondrial proteins, supporting the role of KL on mitochondrial functions. Western blot analyses of the p-AMPK (Thr172) revealed a significant decrease in the expression of this protein in the RPE/retina of *Kl*^-/-^ mice as compared to that of WT mice. AMPK regulates autophagy, which is crucial for cellular waste elimination and energy production during starvation. Our data are supported by a study showing that the restoration of *Kl* can promote autophagy in cells [69]. These observations are of crucial importance since we have previously showed reduced AMPK activity in the RPE of AMD donor eyes [53].

AMPK can also phosphorylate and induce PGC-1α transcription and activity [70,71]. Our data demonstrated a decrease in the PGC-1α protein levels in the RPE/retina of *Kl*^-/-^ mice as compared to those of WT mice. PGC-1α is a master regulator of mitochondrial biogenesis and, thus, could affect mitochondrial function. Our analyses also revealed a reduction in the DRP1 protein levels in the RPE/retina of *Kl*^-/-^ mice as compared to those of WT mice. DRP1 is crucial for mitochondrial fission, and the isolation of damaged mitochondria for degradation [72]. The preservation of mitochondrial integrity and function is vital for cell survival, especially in non-dividing cells that have a high energy demand, such as the RPE. Consequently, reduced PGC-1α and DRP1 protein levels in the RPE/ retina of *Kl*^-/-^ mice could contribute to elevated cell death and apoptosis. mTOR has been shown to induce metabolic switches and degeneration in the RPE [59]. Previously, we showed an overactive mTOR pathway in the RPE of AMD donors [53]. An analysis of mTOR protein levels revealed increased total mTOR and phosphorylated mTOR in the RPE/retina of *Kl*^-/-^ mice compared to those of WT mice. These observations indicate that the lack of KL protein will negatively affect proteins responsible for mitochondrial biogenesis and fission, and will trigger an overactive m-TOR pathway in the RPE/retina of *Kl*^-/-^ mice. Complex I is the first enzyme of the mitochondria respiratory chain and the major entry point for electrons, thus proposed as the rate-limiting step in respiration, and plays a crucial role in energy metabolism [73]. Our analyses of Complex I activity revealed a significant reduction in its activity in the RPE/retina of *Kl*^-/-^ mice as compared to that of WT mice. The mtDNA encodes 13 core proteins, which confer normal functionality in mitochondrial energy metabolism [74]. The alteration of mtDNA could induce mitochondrial dysfunction and degenerative diseases, including age-related macular degeneration (AMD). Studies have shown that mtDNA damage is found in the RPE and macula region of AMD donor eyes [75]. We have also shown dysfunctional and disintegrated mitochondria in the RPE of AMD donor eyes [76]. Our data revealed reduced mtDNA copy numbers in the RPE/retina of *Kl*^-/-^ mice as compared to those of WT mice, indicative of mitochondrial dysfunction. Damaged mitochondria can induce increased levels of ROS [77]. We measured the ROS levels in the RPE/retina of *Kl*^-/-^ and WT mice and found increased ROS levels in the RPE/retina of *Kl*^-/-^mice. The gene expression analyses revealed the regulatory effect of KL on *PGC-1α*, the downstream gene *ACADM*, fission and fusion genes and respiratory Complex I subunit genes. The expression of all the above genes was significantly reduced in the RPE/retina of *Kl*^-/-^as compared to that of the WT mice. These observations further support that KL protein is required for mitochondrial normal activity and scavenging ROS in the RPE/retina. To support our in vivo observations, and further confirm the role of KL on regulating RPE mitochondrial activity and cell viability, we used the CRISPR-Cas9 gene editing to knockout the *KL* gene in the ARPE19, a human RPE cell line. Dox-inducible ARPE19-*KL* KO cells revealed reduced cell viability under oxidative stress conditions as compared to that of the control cells. Interestingly, PGC1α protein was reduced in the ARPE19-*KL* KO cells as compared to that of the control, supporting the regulatory effect of KL on the PGC-1α pathway. As expected, the ARPE19-*KL* KO cells also showed a reduced Complex I activity and mtDNA copy numbers as compared to those of the control cells. In addition, the gene expression levels of *PGC-1α, ACADM, DRP1, OPA1,* and the Complex I subunits were reduced in ARPE19-*KL* KO as compared to those of the control cells. These observations further support the role of KL on mitochondrial biogenesis as well as activity and cell viability in the RPE. 

## 5. Conclusions

**Figure 7:**
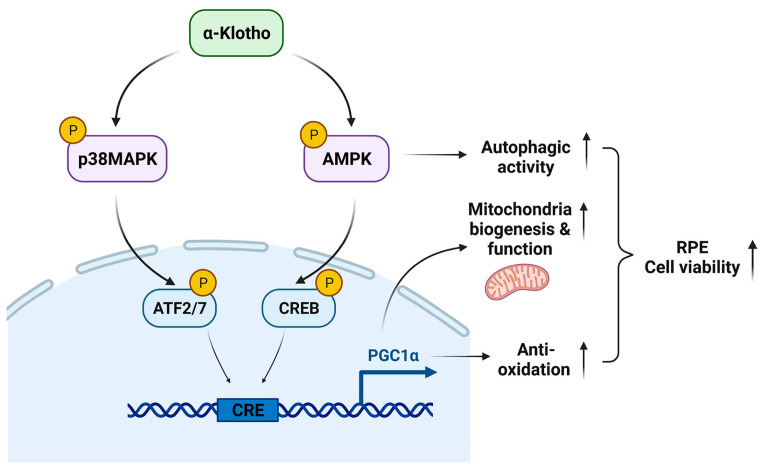
The proposed model of KL action in RPE.

Our data demonstrate the underlying mechanisms by which KL regulates the RPE and retinal homeostasis. KL influences cell viability by the induction of mitochondrial biogenesis and activity, and scavenging ROS in the RPE. Our observations also indicate that KL regulates the above functions by activating the AMPK/PGC-1α pathway in RPE cells. A recapitulative image of KL action on the RPE/retina is shown in Figure 7. Our data open avenues for developing new treatment strategies for age-related macular degeneration.

KL phosphorylates and activates AMPK and p38MAPK, which in turn phosphorylate CREB and ATF2/7, respectively. Both CREB and ATF2 act on the same CRE site and induce the transcriptional activation of PGC-1α. Thus, KL promotes RPE cell viability by increasing mitochondrial biogenesis and functions as well as scavenging the ROS by activating the AMPK/PGC-1α pathway.

## Data Availability

The data are provided in the manuscript. Proteomic raw data can be shared upon request.

## Supplementary Materials

The following supporting information can be downloaded at: https://www.mdpi.com/article/10.3390/antiox12020385/s1. Figure S1. Identification of ARPE19 CRISPR/Cas9 *KL* KO cells and verification of the efficiency of *KL* inhibition with different sgRNAs. (A) ARPE19-*KL* KO cells were incubated with 2 μM Doxycycline (DOX) for 48h to test the eGFP, which confirms the expression of Cas9. (B) ARPE19 cells were transfected with CRISPR/Cas9-*KL* sgRNAs vectors and selected by 2 μg/mL puromycin for 2 weeks, followed by incubation with 2 μM Doxycycline for 48h to induce the Cas9-eGFP expression. Flow cytometry was performed to analyze the ratio of Cas9-eGFP cells. (C,D) KL protein levels in Scramble control group and 2 different sgRNA groups were analyzed by Western Blots. Compared with control, KL protein was inhibited ~31% by sgRNA1 and 66% by sgRNA2. The relative densities of the bands were determined using ImageJ software. The Unpaired *t*-test was performed using GraphPad Prism 8.3.0. Graph represents mean ± SEM, n = 3, ** *p* ≤ 0.01. Table S1. Primers used for qPCR analyses. Table S2. Antibodies used for Western blot analyses
