# Supplementary material for: The Anti-Aging Hormone Klotho Promotes Retinal Pigment Epithelium Cell Viability and Metabolism by Activating the AMPK/PGC-1α Pathway
Source: Antioxidants (Basel). 2023 Feb 5;12(2):385. doi: 10.3390/antiox12020385 (PMC9952846; doi:10.3390/antiox12020385)
Supplement: Supplementary file 1 [file antioxidants-12-00385-s001.zip › antioxidants-2164285-supplementary.pdf]

Supplementary Data

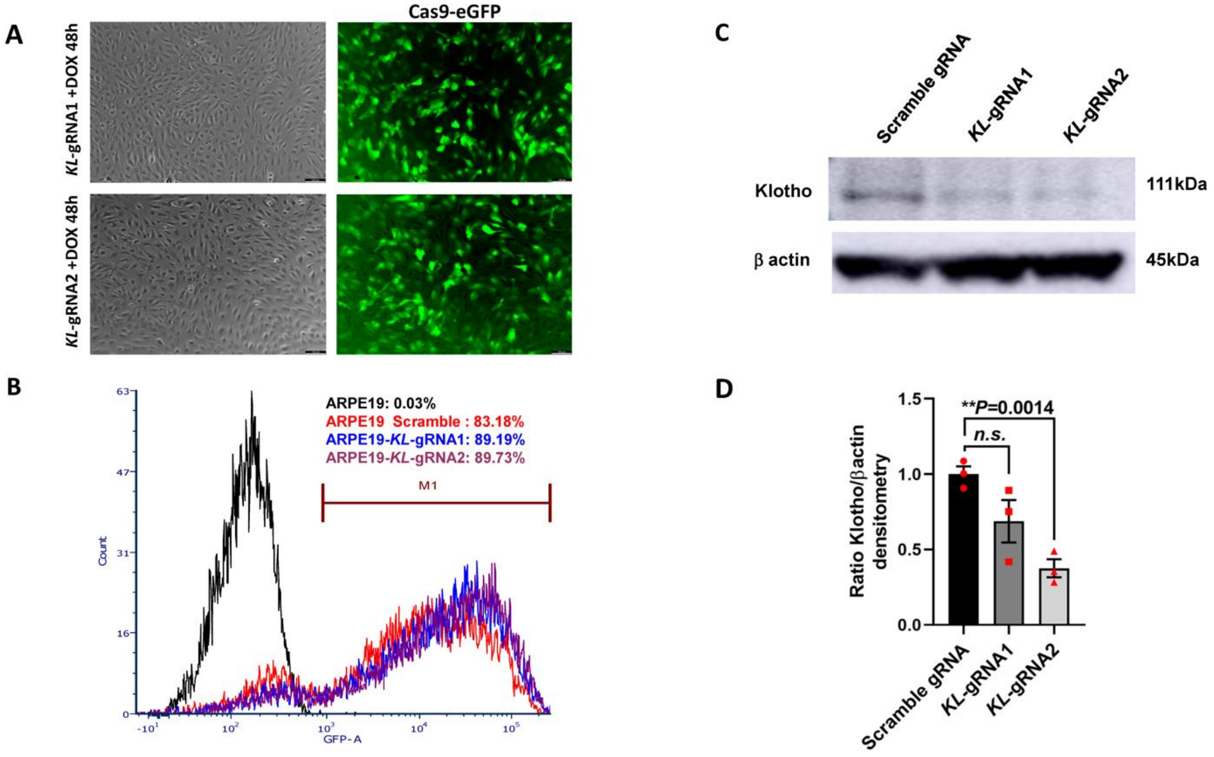

Figure S1. Identification of ARPE19 CRISPR/Cas9 *KL* KO cells and verification of the efficiency of *KL* inhibition with different sgRNAs

(**A**) ARPE19-*KL* KO cells were incubated with 2μM Doxycycline (DOX) for 48h to test the eGFP, which confirms the expression of Cas9. (**B**) ARPE19 cells were transfected with CRISPR/Cas9-*KL* sgRNAs vectors and selected by 2μg/ml puromycin for 2 weeks, followed by incubation with 2μM Doxycycline for 48h to induce the Cas9-eGFP expression. Flow cytometry was performed to analyze the ratio of Cas9-eGFP cells. (**C**,**D**) KL protein levels in Scramble control group and 2 different sgRNA groups were analyzed by Western Blots. Compared with control, KL protein was inhibited ~ 31% by sgRNA1 and 66% by sgRNA2. The relative densities of the bands were determined

using ImageJ software. The Unpaired *t*-test was performed using GraphPad Prism 8.3.0. Graph represents mean±SEM, n=3, ***P*≤0.01.

Table S1. Primers used for qPCR analyses.

Primers	Primer Sequence (5'-3')
human *PGC-1a* FOR<br>human *PGC-1a* REV	TCTGAGTCTGTATGGAGTGACAT<br>CCAAGTCGTTCACATCTAGTTCA
human *DRP1* FOR<br>human *DRP1* REV	TTTGACACTTGTGGATTTGCCA<br>AGTGACAGCGAGGATAATGGA
human *ACADM* FOR<br>human *ACADM* REV	ACAGGGGTTCAGACTGCTATT<br>TCCTCCGTTGGTTATCCACAT
human *NDUFS2* FOR<br>human *NDUFS2* REV	GCTGTTATGTACCCAAGCAAAGA<br>TCCCCACTCAATTCCATCACT
human *NDUFS8* FOR<br>human *NDUFS8* REV	CCATCAACTACCCGTTCGAGA<br>CCGCAGTAGATGCACTTGG
human *NDUFA8* FOR<br>human *NDUFA8* REV	CCCAACAAGGAGTTTATGCTCT<br>CACAGTGACGTTTTATCTGCCT
human *NDUFB10* FOR<br>human *NDUFB10* REV	AGCCCAATCCCATCGTCTACA<br>GCTGCCGCTCTATAAATTCTCT
human *OPA1* FOR<br>human *OPA1* REV	TGTGAGGTCTGCCAGTCTTTA<br>TGTCCTTAATTGGGGTCGTTG
human *ND1* FOR<br>human *ND1* REV	CCTAGCCGTTTACTCAATCCT<br>TGATGGCTAGGGTGACTTCAT
human *ACTB* FOR<br>human *ACTB* REV	CATCTCTTGCTCGAAGTCCA<br>ATCATGTTTGAGACCTTCAACA

human *GAPDH* FOR<br>human *GAPDH* REV	GGAGCGAGATCCCTCCAAAAT<br>GGCTGTTGTCATACTTCTCATGG
Mouse *Pgc1a* FOR<br>Mouse *Pgc1a* REV	AGCCGTGACCACTGACAACGAG<br>GCTGCATGGTTCTGAGTGCTAAA
Mouse *Gapdh* FOR<br>Mouse *Gapdh* REV	AGACAGCCGCATCTTCTTGT<br>AATCTCCACTTTGCCACTGC
Mouse *Acadm* FOR<br>Mouse *Acadm* REV	AACACAACACTCGAAAGCGG<br>TTCTGCTGTTCCGTCAACTCA
Mouse *Drp1* FOR<br>Mouse *Drp1* REV	CAAGGTTTTCTCGCCCAACG<br>CTGCCCTTACCATCTGGATCTA
Mouse *Ndufs2* FOR<br>Mouse *Ndufs2* REV	CAGCCAGATATTGAATGGGCA<br>TGTTGGTCACCGCTTTTTCCT
Mouse *Ndufs8* FOR<br>Mouse *Ndufs8* REV	AGTGGCGGCAACGTACAAG<br>TCGAAAGAGGTAACTTAGGGTCA
Mouse *Ndufa8* FOR<br>Mouse *Ndufa8* REV	GGAGCTGCCAACTCTGGAAG<br>CCAGCGGCACAGCATAAAC
Mouse *Ndufb10* FOR<br>Mouse *Ndufb10* REV	CTCCCTAACCCCATCACCTAC<br>TGGCACTCGACGGTACTGT
Mouse *Opa1* FOR<br>Mouse *Opa1* REV	TGGAAAATGGTTCGAGAGTCAG<br>CATTCCGTCTCTAGGTTAAAGCG
Mouse *Nd1* FOR<br>Mouse *Nd1* REV	CTCTTATCCACGCTTCCGTTACG<br>GATGGTGGTACTCCCGCTGTA
Mouse *H19* FOR<br>Mouse *H19* REV	GTACCCACCTGTCGTCC<br>GTCCACGAGACCAATGACTG

Table S2. Antibodies used for Western blot analyses

Antibody	Company	Catalog Number	Dilution
AMPK alpha Rabbit	Cell Signaling Technology	5832S	1:1000
p-AMPK alpha Rabbit	Cell Signaling Technology	2535S	1:1000
ATF2/ATF7 Rabbit	Cell Signaling Technology	82870S	1:1000
p- ATF2/ATF7 Rabbit	Cell Signaling Technology	24329S	1:1000
p38 MAPK Rabbit	Cell Signaling Technology	8690S	1:1000
p-p38 MAPK Rabbit	Cell Signaling Technology	9215S	1:1000
CREB Rabbit	Cell Signaling Technology	9197S	1:1000
p- CREB Rabbit	Cell Signaling Technology	9198S	1:1000
mTOR Rabbit	Cell Signaling Technology	2972S	1:1000
p-mTOR Rabbit	Cell Signaling Technology	2974S	1:1000
DRP1 Rabbit	Cell Signaling Technology	8570S	1:1000
GAPDH Rabbit	Cell Signaling Technology	5174S	1:8000
β actin Rabbit	Cell Signaling Technology	8457S	1:5000-1:30000
Goat anti-Rabbit IgG	Jackson ImmunoResearch	111-035-144	1:4000
Anti-PGC1a (Rabbit)	Millipore	AB3242	1:1000
Anti-KLOTHO (Rabbit)	Sigma Aldrich	SAB3500604	3μg/ml
